# Piperacillin/Tazobactam Co-Delivery by Micellar Ionic Conjugate Systems Carrying Pharmaceutical Anions and Encapsulated Drug

**DOI:** 10.3390/pharmaceutics16020198

**Published:** 2024-01-30

**Authors:** Katarzyna Niesyto, Aleksy Mazur, Dorota Neugebauer

**Affiliations:** Department of Physical Chemistry and Technology of Polymers, Faculty of Chemistry, Silesian University of Technology, 44-100 Gliwice, Poland; katarzyna.niesyto@polsl.pl (K.N.); aleksy.mazur@polsl.pl (A.M.)

**Keywords:** co-delivery systems, PIL, piperacillin, tazobactam, encapsulation

## Abstract

Previously obtained amphiphilic graft copolymers based on [2-(methacryloyloxy)ethyl]trimethylammonium chloride (TMAMA) ionic liquid were used as the matrices of three types of nanocarriers, i.e., conjugates with ionic piperacillin (PIP) and micelles with tazobactam (TAZ), which represented single systems, and dual systems bearing PIP anions and encapsulated TAZ for co-delivery. The exchange of Cl anions in TMAMA units with PIP ones resulted in a yield of 45.6–72.7 mol.%. The self-assembling properties were confirmed by the critical micelle concentration (CMC), which, after ion exchange, increased significantly (from 0.011–0.020 mg/mL to 0.041–0.073 mg/mL). The amphiphilic properties were beneficial for TAZ encapsulation to reach drug loading contents (DLCs) in the ranges of 37.2–69.5 mol.% and 50.4–80.4 mol.% and to form particles with sizes of 97–319 nm and 24–192 nm in the single and dual systems, respectively. In vitro studies indicated that the ionically conjugated drug (PIP) was released in quantities of 66–81% (7.8–15.0 μg/mL) from single-drug systems and 21–25% (2.6–3.9 μg/mL) from dual-drug systems. The release of encapsulated TAZ was more efficient, achieving 47–98% (7.5–9.0 μg/mL) release from the single systems and 47–69% (9.6–10.4 μg/mL) release from the dual ones. Basic cytotoxicity studies showed non-toxicity of the polymer matrices, while the introduction of the selected drugs induced cytotoxicity against normal human bronchial epithelial cells (BEAS-2B) with the increase in concentration.

## 1. Introduction

Versatility of application, as well as the possibility of designing drug carriers, has resulted in drug delivery systems (DDSs) gaining high interest among polymer scientists. In past years, DDSs have been studied to improve the healing effect and potential of drugs [1,2,3,4,5]. There are a few factors, i.e., toxicity, non-stability, and poor solubility of the drugs, that limit the capacity of pharmaceuticals. Functional polymers, in addition to being able to transport the drug to the desired place, perform various tasks, for example, enhancing bioavailability or bioactivity, drug protection from undesired external factors, and drug decomposition, or even enhancing the solubility of the drug [6,7,8]. The composition of the polymer that constitutes the matrix is fundamental to obtaining the desired carrier with appropriate properties. There are many types of carriers, and those based on poly(ionic liquids) (PILs) deserve special attention due to the unique properties of ionic liquids.

PILs comprise ions in their structure and have the specific properties of ionic liquids (ILs), such as high thermal stability, high solvating power, and low vapor pressure [9,10,11]. Moreover, PILs demonstrate unrepeatable properties due to the combined architecture of the polymer and ILs [12,13]. From the DDS design and pharmacological points of view, ILs are useful in the development of new pharmaceutical agents and in the organic synthesis of pharmaceutics as green solvents, which facilitates the purification and isolation of pharmaceutical compounds [14,15,16,17]. Moreover, ILs and PILs can affect the solubility of ionically bounded drugs [18,19,20,21]; they can serve as prodrugs [22,23,24,25]; and by properly selecting the polymer chain and the pharmaceutical counterion, the fine tuning of their properties is possible [26,27]. Many PILs have non-toxic, biocompatible, and self-assembly properties, which give them the possibility of being matrices of nanocarriers in DDSs, as has been reported, for example, for imidazolium PIL-based polymers [28]. Spherical nanoparticles prepared from an amphiphilic block copolymer with PIL units have been studied in doxorubicin delivery [29]. The same drug has been successfully encapsulated and delivered by phosphonium PIL-based nanoparticles [30]. On the other hand, the more sophisticated structure of cholinium PIL-based graft copolymers is designed to carry *p*-aminosalicylate, clavulanate [31], cloxacillin [32], and fusidate [33].

One of the main problems in chemotherapy is overcoming drug resistance and improving therapy. Therefore, using dual-drug systems for combination therapy that includes various pharmaceutics with synergistic action has become a desired method [34,35,36]. There are few diverse approaches to obtaining dual-drug systems, but those based on PILs are still not well known and need extensive investigations. In the literature, there are only a few examples of the use of polymers based on ionic liquids for the simultaneous release of different drugs. For example, polymeric multi-branched IL-based chitosan nanocarriers for simultaneous delivery of doxorubicin and methotrexate have been reported [37]. Dual systems have also been studied by our group; the polymer carriers based on choline ionic liquid demonstrated successful delivery of salicylate and erythromycin [38], fusidate and rifampicin [33], and fusidate and cloxacillin [32] as pairs of drugs with a synergistic effect.

**Scheme 1:**
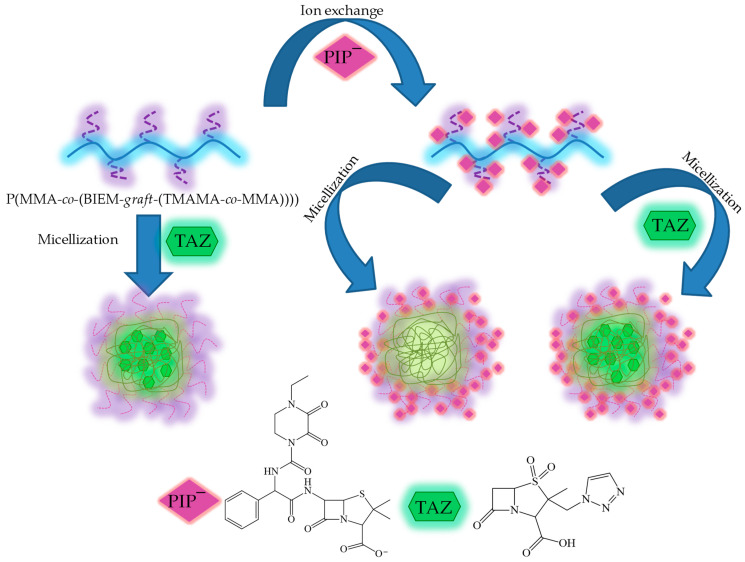
Schematic routes of single- and double-drug systems with piperacillin anions and tazobactam as model drugs.

In this paper, we present a study on well-defined graft copolymers which incorporate water-soluble [2-(methacryloyloxy)ethyl]trimethylammonium chloride (TMAMA) IL units in their side chains, as outlined in reference [31]. These copolymers serve as matrices for the development of single- and dual-drug delivery systems. The biological nature of the polymers was generated by introducing TMAMA as the derivative of choline, which has a complex role in living organisms, including the synthesis of neurotransmitters, the transformation of betaine for methyl-group metabolism, and the synthesis of cell membrane components and lipoproteins. Due to the presence of ionic moieties in the side chains, the ion exchange of chloride with ionic drugs was possible, thereby modifying the polymer. On the other hand, the amphiphilic polymers were also verified in terms of the capability of non-ionic drug encapsulation. For this purpose, piperacillin (PIP) in ionic form was selected as a model drug to obtain ionic polymer–drug conjugates, whereas tazobactam (TAZ) was the second type of model drug with a non-ionic nature beneficial for loading in the formed micelles. Recently, our group reported polymer–PIP-based ionic conjugates with a linear topology [39], but the graft copolymers with a specific structure formed more stable self-assembled nanostructures than their linear analogs, providing different drug release profiles. TAZ and PIP are commonly applied as a standard formulation of drugs with a broad spectrum of antibacterial action, also known as the commercial product Zosyn^®^. So far, PIP/TAZ have been co-encapsulated in micelles [40], incorporated into hydrogels [41], and mixed with nanocomposites [42]. In our studies, these two drugs were introduced via different bonding types to diverge their co-release rate, whereby the encapsulated drug loaded through physical interactions was more readily available for drug release than the ionically conjugated drug. Therefore, to show the influence of drug nature and polymer matrix composition, as well as drug connection type, three types of carriers were examined, i.e., ionic conjugates (PIP) and micelles (TAZ) as single DDSs and micelle-forming polymer–drug conjugates carrying TAZ and PIP with synergistic action as a dual system (Scheme 1). Their potential was verified through the determination of drug contents and/or loading efficiency, kinetic profiles of in vitro drug release in phosphate-buffered saline (PBS) imitating human fluids (pH 7.4, 37 °C), and cytotoxicity against the BEAS-2B cell line using an MTT assay.

## 2. Materials and Methods

Graft copolymers (GP1–GP3) based on PILs with choline cations and chloride counterions (Table 1) were synthesized through controlled radical polymerization and characterized according to previously described procedures [31,43]. Sodium piperacillin (NaPIP; 99%) and tazobactam (TAZ; 94%) were purchased from Alfa Aesar (Warsaw, Poland) and used without prior purification. Methanol was obtained from Chempur (Piekary Śląskie, Poland). Phosphate-buffered saline (PBS) was obtained from Sigma-Aldrch (Poznań, Polska). DMEM-F12 medium and 3-(4,5-dimethyl-thiazol-2-yl)-2,5-diphenyltetrazolium bromide (MTT) were received from Aldrich (Poznań, Poland). Human bronchial epithelial cells (BEAS-2B) were purchased from ATCC (Cat# ATCC^®^ CRL-9609; Manassas, VA, USA).

### 2.1. Synthesis of Ionic Conjugates Bearing PIP Anions (Example of GP1_PIP^−^)

Copolymer GP1 (21 mg, including 0.05 mmol TMAMA units) was dissolved in methanol (1 mL). Subsequently, pharmaceutical PIP sodium salt (29.9 mg, 0.05 mmol) was added to the mixture in equimolar ratio to the TMAMA units in the polymer chain. The reaction was carried out for 48 h at room temperature. Then, the polymer system was dried under reduced pressure, resulting in the formation of conjugate GP1_PIP^−^.

### 2.2. Encapsulation of TAZ

A graft copolymer bearing chloride counterions (20 mg) and TAZ (20 mg) was dissolved in methanol (2 mL). After complete dissolution, a two-fold excess of deionized water was added to the mixture and stirred for 24 h. Subsequently, the organic solvent was evaporated. The resulting aqueous fraction was then lyophilized, yielding the solid product.

The same procedure was used to obtain dual systems containing two types of drugs, PIP and TAZ. In this case, the ionic PIP-based polymer conjugate (20 mg) was dissolved with TAZ (20 mg) in methanol.

### 2.3. In Vitro Drug Release Studies

Single-/dual-drug systems (1.0 mg) were dissolved to achieve a concentration of 1 mg/mL in PBS (pH = 7.4). A volume of 1 mL of the obtained mixture was introduced to a dialysis membrane bag (MWCO = 3.5 kDa) and placed into a glass vial containing 45 mL of PBS. The drug release experiment was conducted at a temperature of 37 °C under constant stirring. Samples of the buffer solution (0.5 mL) were taken and mixed with an equal volume of methanol to determine the concentration of released drug using UV-Vis spectroscopy, measuring absorbance at λ = 277 nm for PIP anions and λ = 210 nm for TAZ. The data obtained from the measurements are presented in release profiles as means ± SDs.

### 2.4. Cell Growth and MTT Cytotoxicity Assay

Cells were cultured in a DMEM-F12 medium in sterile culture bottles, characterized by 75 cm^2^ of culture area, supplemented with 10% (*v/v*) FBS at 37 °C in the incubator (humidified atmosphere with 5% CO_2_). The cell cultures were placed in a 96-well plate for MTT tests with 10,000 cells per well. A total of 10,000 cells were placed into 96-well plates in 0.2 mL of medium 24 h before polymer systems were added.

Control samples were prepared in the first row and outer columns of wells. Dilutions of the tested compounds (3.125–100 μg/mL) were prepared in the left wells (0.1 mL). The treated and control cells were then incubated for 72 h under standard conditions. Subsequently, the solutions were removed, and 50 μL of MTT solution (0.5 mg/mL in RPMI 1640 without phenol red) was added into each well. After 1–2 h of incubation, the MTT solution was aspirated. The created formazan crystals were dissolved in 75 μL of isopropanol:HCl mixture (*v*/*v* = 1:0.04). Cytotoxicity was evaluated by measuring the absorbance of the formazan product at 570 nm using a microplate reader. Measurements were repeated three times (six technical repetitions for each concentration). The results were presented as the percentage fraction of the control. Cell viability monitoring and confluence analysis were performed using a live cell analyzer. After 72 h of incubation, microscopic images of both treated and untreated cells were captured. Data obtained from measurements are presented as means ± SDs.

### 2.5. Characterization

Critical micelle concentration (CMC) was determined by measuring interfacial tension (IFT) using the pendant drop method on a goniometer (OCA 15EC; DataPhysics, Filderstadt, Germany). For this purpose, a series of aqueous polymer solutions (0.0006–0.15 mg/mL) were prepared. The goniometer was also used for evaluating the contact angle (CA) using the sessile drop method. The polymer solution in methanol (0.3 mg/mL) was spin-coated on a thin glass plate. Next, 4 μL of deionized water was dispensed onto the polymer layer; then, the CA value was measured. The data were collected and processed with SCA20_U software 5.0.38. The hydrodynamic diameter (D_h_) of particles and the polydispersity index (PDI) were measured with dynamic light scattering (DLS) using a nanoparticle analyzer, NANOTRAC Flex (Microtrac Retsch GmbH, Haan, Germany; Dimensions LS software 1.1.0.), equipped with an external “dip-in” probe with 180° backscattering. Samples were placed and measured in 1.5 mL glass vials after dilution with deionized water (1.0 mg/mL) at 25 °C. Each measurement was repeated three times to create an average value. Ultraviolet–visible light spectroscopy (UV-Vis; spectrometer Evolution 300; Thermo Fisher Scientific, Waltham, MA, USA) was used to determine the ionic drug content (DC) or the non-ionic drug loading content (DLC), as well as the amount of drug released during in vitro studies in PBS with the addition of methanol. The calculations were based on the estimated absorbance of detected pharmaceuticals at proper wavelengths (277 nm for PIP and 210 nm for TAZ) to determine their concentrations using the calibration curve equations using standard formulas [31]. The measurements were carried out in quartz cuvettes. Viability monitoring and confluence analysis were performed using a live cell analyzer (JuLI™ Br; NanoEnTek Inc., Seoul, Korea). The cytotoxicity according to the MTT test was evaluated by measuring the absorbance of formazan product at 570 nm with the use of a microplate reader (Epoch; BioTek, Winooski, VT, USA).

## 3. Results and Discussion

The graft polymers (GP1–GP3) were composed of methyl methacrylate and 2-(2-bromoisobutyryloxy)ethyl methacrylate copolymers as the main chain, decorated by [2-(methacryloyloxy)ethyl]trimethylammonium chloride and methyl methacrylate copolymers as the grafts (P(MMA-*co*-(BIEM-*graft*-(TMAMA-*co*-MMA)))) [31]. The copolymers varied in terms of the number of side chains (n_sc_ = 48–133), grafting degree (DG = 26–46%), polymerization degree of side chains (DP_sc_ = 28–65), and content of TMAMA units (F_TMAMA_ = 18–36 mol.%) (Table 1). These designed graft copolymers with advanced structures are dedicated to antibacterial treatment, including combination therapy. 

**Figure 1:**
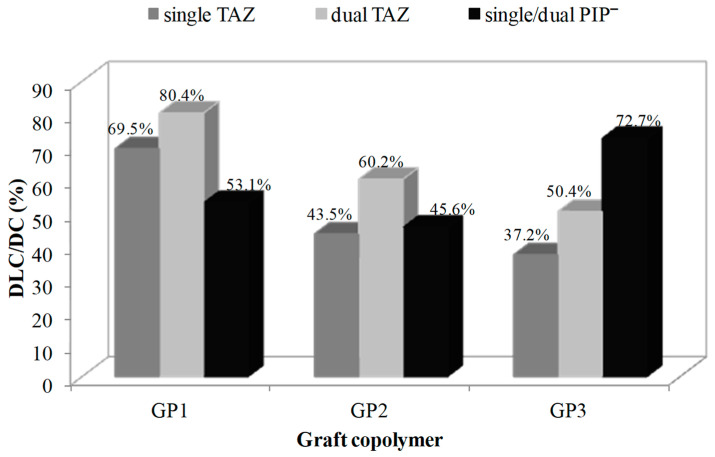
Drug loading contents of TAZ vs. drug contents of PIP anions in single and dual polymeric systems.

The self-assembling characteristics of the graft copolymers with Cl anions have already been evaluated with critical micelle concentration values (CMC; Table 2) ranging from 0.011 to 0.020 mg/mL [31]. The amphiphilicity of these copolymers allowed for non-ionic drug encapsulation, resulting in micelles as a single-drug delivery system (Scheme 1). To assess the loading capability, tazobactam (TAZ) was used as a model drug. TAZ is a penicillanic acid derivative with a β-lactam antibiotic structure which works as a β-lactamase inhibitor commonly used with β-lactam antibiotics, such as piperacillin, to protect them from bacterial destruction [44]. The copolymer structure directly influenced the efficiency of encapsulation (Figure 1). In the case of copolymer GP1, with a lower number of side chains represented by a lower degree of grafting, encapsulation proceeded to the highest degree, leading to a nearly 70% drug loading in the micelles. For copolymers GP2 and GP3, with a higher number of side chains and a higher degree of grafting, encapsulation was less effective (DLC = 43.5% and 37.2%, respectively). Additionally, it was observed that the degree of encapsulation increased with the fraction of TMAMA units. Thus, both a high fraction of TMAMA units (as the hydrophilic fraction) and a lower density of side chains may have a beneficial effect on TAZ encapsulation inside the micelles formed by choline-based copolymers with Cl counterions.

Subsequently, due to the presence of TMAMA units in the side chains of graft copolymers, the exchange reaction from chloride to pharmaceutical anions was possible. Consequently, piperacillin (PIP) anions were introduced into the polymer structure, and polymer–drug ionic conjugates were obtained as a second series of single DDSs (Scheme 1). Piperacillin, a β-lactam antibiotic with a broad spectrum of action, was used as the model drug in this case. Its mechanism focuses on blocking bacterial cell wall biosynthesis (transpeptidation) due to the structural similarity to the natural substances used by bacteria to build the cell wall. The drug content (DC; Figure 1), determined with the UV-Vis method, suggested that the exchange between chloride and PIP ions was the most effective in the case of copolymer GP3 (~73%), characterized by the highest grafting density and the longest side chains. Lower DC values were detected for GP1 (53%) and GP2 (45%), characterized by doubly shorter grafts and doubly higher content of ionic fraction (above 35 mol.%). It is possible that the high amount of MMA in the side chains led to a lower packing of the copolymer matrix, resulting in a looser distribution of ionic TMAMA units, facilitating access to them.

Following ion exchange, the amphiphilic properties of graft copolymers needed verification for their use in further encapsulation of a second drug. The CMC values of the ionic PIP polymer conjugates were determined using the goniometric method, where the intersection of two straight lines on the interfacial tension (IFT) versus the negative logarithm of concentration (−logC) plot was employed as the standard procedure. In comparison to the initial graft copolymers with Cl anions, the CMCs after conjugation with PIP significantly increased, yielding values in the range of 0.041–0.073 mg/mL (Table 1). However, the conjugates were still able to form micelles. Therefore, drug encapsulation was possible, and they could be employed as matrices for dual-drug systems, representing a third type of the studied carriers.

**Figure 2:**
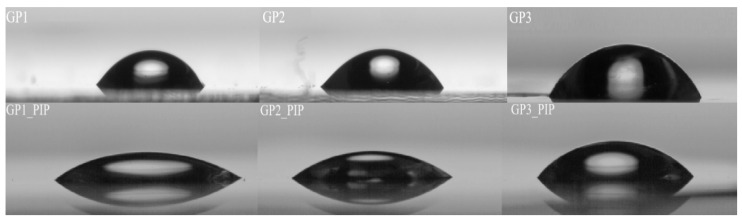
Screen shots of contact angles on polymer films of GP1–GP3 vs. GP1_PIP–GP3_PIP measured using the goniometric method.

The polymer characteristics were also examined to determine the surface wettability of PIP conjugate films. For this purpose, solutions of conjugates at a concentration 0.3 mg/mL in methanol were prepared. The glass plates were deeply cleaned for thin polymeric layer application. The spin-coating method was used to obtain a homogeneous polymer layer. Then, the goniometer was applied to measure the contact angle (CA) using the sessile drop method. After anion exchange between chloride anions and PIP ones, the CA values decreased as follows: GP1: 56.3° vs. 36.1°; GP2: 48.9° vs. 35.5°. On the contrary, we did not notice any changes in the case of GP3 (Table 2). The highest reduction was observed in GP1_PIP, which was also characterized by the highest increase in CMC. Considering CMC and CA values, it can be concluded that conjugation with a chosen ionic drug induced an enhancement in the hydrophilicity of the polymer systems. Additionally, they became more hydrophilic with the increase in the content of ionic fraction, which is an opposite correlation with respect to the chloride-based systems. The photos from goniometric measurements are present in Figure 2.

The particle sizes of the single- and dual-drug systems were assessed with dynamic light scattering (DLS) measurements (Table 3, Figure S1). The encapsulation of TAZ in chloride-based copolymers created monodisperse particles for GP1 and GP3 ranging between 319 nm and 247 nm, while in GP2, the prevailing fraction (>40%) demonstrated particles of 97 nm. A higher grafting degree of copolymers resulted in the aggregation of nanoparticles, especially for G2 (~10%) and insignificantly for G3. In the case of PIP polymer conjugates, the predominant fraction (>68%) consisted of nanoparticles in the size range of 20–31 nm. The π-stacking interaction, attributed to PIP conjugation with the polymer containing the highest TMAMA fraction, was responsible for the attraction of GP1 polymer assemblies (>1700 nm, 10%). Dual-drug systems showed a tendency for the formation of two prevailing fractions in the size range of 24–192 nm, indicating smaller particles than TAZ-encapsulating single-drug systems and relatively similar to analogous PIP conjugates. Generally, the longest side chains and the highest DC of PIP in the GP3 sample favored a repulsion effect, leading to larger nanoparticles as small aggregates in a significant fraction (451 nm in 32% for single systems and 770 nm in 46% for the dual systems).

**Figure 3:**
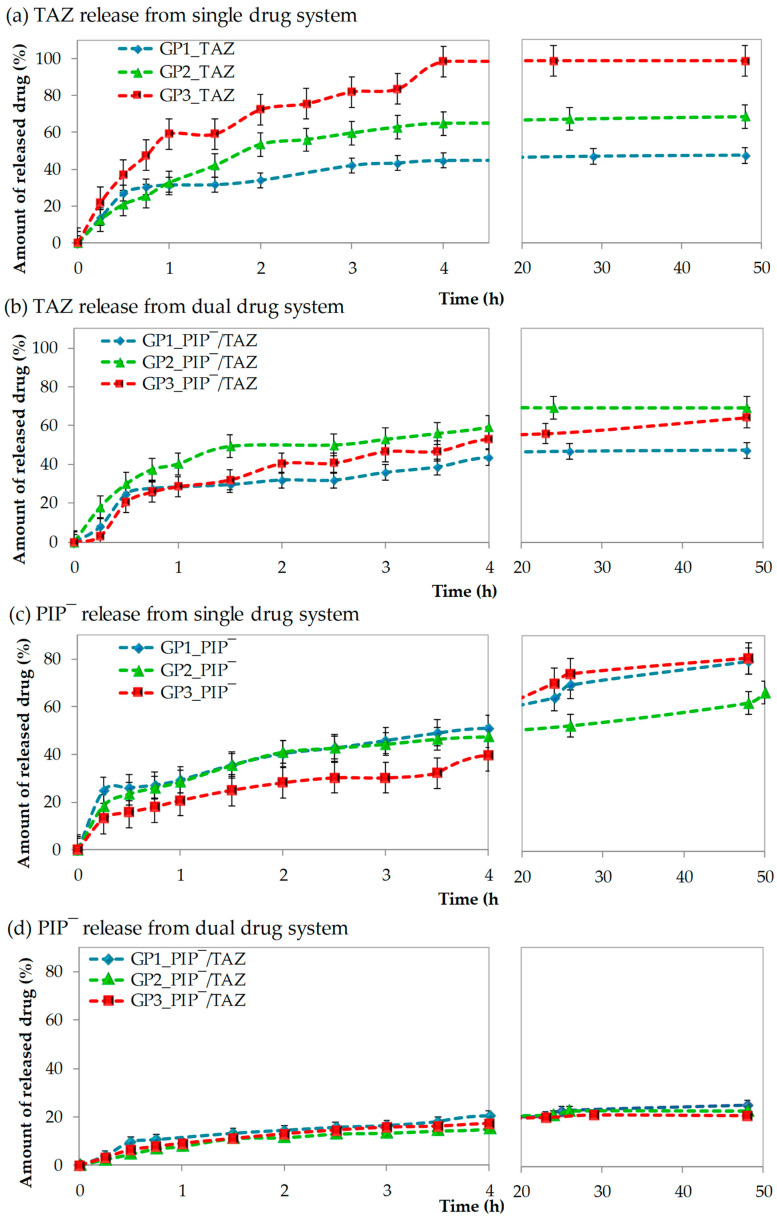
Release profiles of TAZ (**a**,**b**) and PIP (**c**,**d**) from systems based on graft copolymers.

In vitro drug release from single- and dual-drug systems was conducted at 37 °C in phosphate-buffered saline (PBS; pH = 7.4). The concentration of free drug was monitored within 48 h using UV-Vis spectrometry (Table 4). A similar trend was observed in the case of copolymers GP1 and GP2. In both instances, equivalent amounts of non-ionic TAZ were released from single and dual systems, constituting 47% and ~70%, respectively. This indicates no discernible effect of the conjugated PIP in the polymer matrix (Figure 3a,b). However, considering the different DLC values, which were higher for dual systems, the concentrations of free TAZ exhibited slight variations (GP1: 8.30 μg/mL vs. 9.60 μg/mL; GP2: 7.52 μg/mL vs. 10.41 μg/mL for single and dual systems, respectively). On the other hand, release from the GP3 matrix in a single system progressed to a very high amount of free TAZ (98%), while the presence of PIP anions as the accompanying drug limited the release of encapsulated TAZ to 64%. Despite this, the lower DLC in the micelles than in the double system resulted in a significant concentration of free TAZ (~9 μg/mL). The release of ionic PIP, occurring through exchange with phosphate anions present in the solution, was pronounced for the single system (Figure 3c,d), leading to substantial drug concentrations (7–15 μg/mL). In turn, the presence of encapsulated TAZ in the micellar PIP conjugates significantly restricted the release of ionic PIP to 21–25% (2.6–3.9 μg/mL). In a previous study [25], analogous systems with a different pair of drugs, i.e., ionic fusidate and non-ionic rifampicin, were described. The release was not only influenced by the structure of the polymer matrix, but it could also be limited/improved by the presence of the combined drug and the method of drug introduction (chemical bonding or physical interactions).

The in vitro release of the tested drugs was also analyzed using kinetic models, i.e., the first-order, Higuchi, and Korsmeyer-Peppas model equations (Table 5, Figure S2). The samples did not conform to the zero-order model equation, primarily due to the independence of concentration from drug release. The high correlation coefficients (R^2^), particularly for PIP conjugate systems (R^2^ ≥ 0.90) in the first-order equation, illustrate the time dependence on the percentage of remaining drug. Both single and double systems containing TAZ were characterized by R^2^ values ranging from 0.77 to 0.96, with the lowest value being observed for the GP3 single system. The elevated R^2^ values suggest a good fit, allowing the results to be described by this equation. The Higuchi model equation plots depict the function of the square root of time against cumulative percentage drug release, providing insights into the mechanism of controlled drug diffusion. The drug release profiles of ionic PIP and non-ionic TAZ for all studied systems were linear on the Higuchi plots (R^2^ = 0.89–0.99), indicating diffusion-dependent release. The diffusion of the ionic drug was also assessed using the Korsmeyer–Peppas model equation (M_t_/M = kt^n^). The release exponent (n), determined using this equation, characterizes different release mechanisms. Several single systems (GP1_TAZ, GP1_PIP, GP2_PIP, and GP3_PIP) and TAZ in dual system GP2_PIP^−^/TAZ can be described by a quasi-Fickian process (n ≥ 0.45), while other systems exhibited non-Fickian-type release, indicative of anomalous drug transport (0.45 < n < 0.89).

**Figure 4:**
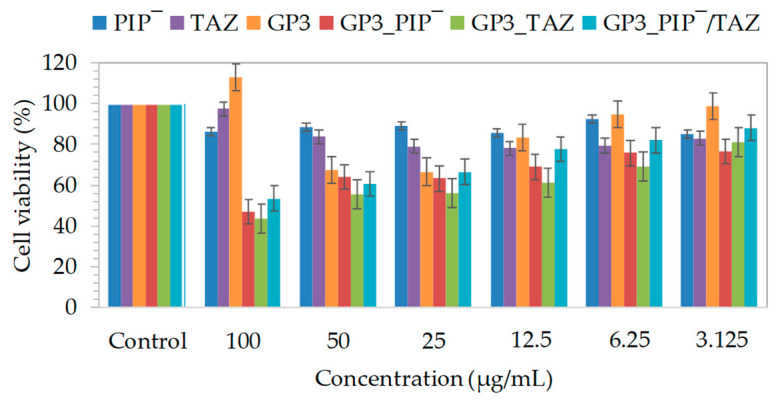
Cell viability of PIP, TAZ, and GP3 bearing PIP^−^ or/and TAZ at different concentrations for treatment of BEAS-2B cell line after 72 h of incubation in comparison to the controls (100%).

**Figure 5:**
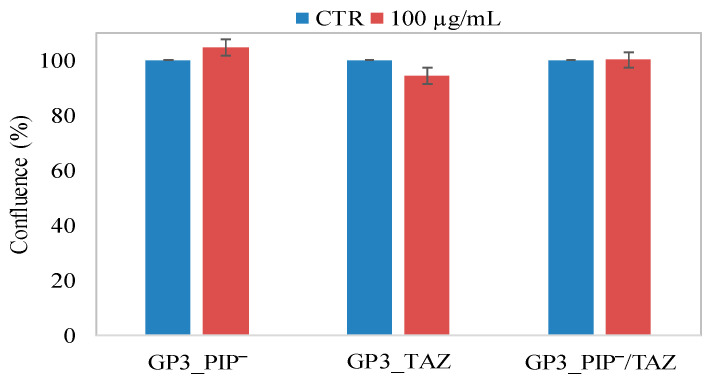
Confluence of BEAS-2B cell lines treated with single and dual systems of GP3 graft polymer for 72 h at the concentration of 100 µg/mL. Results are presented as percent of controls.

**Figure 6:**
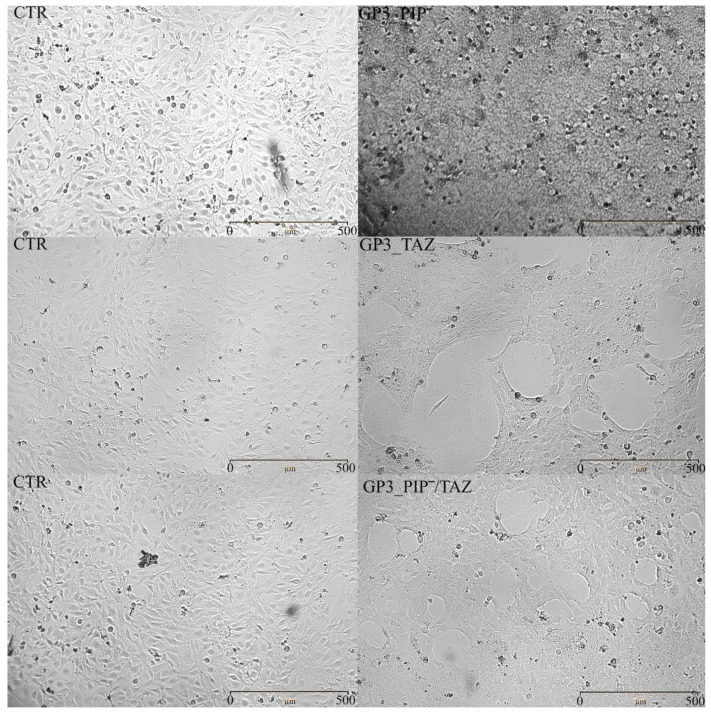
Microscopic images obtained with live cell analyzer of untreated control cells vs. BEAS-2B cells treated with single- or dual-drug delivery graft copolymer systems. The scale bar represents 500 µm.

The previously reported biological studies of graft polymer systems demonstrated selective activity, inducing a negative effect on the tumor adeno-carcinomic human alveolar basal epithelial (A549) cell line, while they did not cause negative changes in normal human bronchial epithelial (BEAS-2B) cells [43]. In this study, co-delivery systems carrying PIP and TAZ were evaluated in BEAS-2B cells to exclude potential cytotoxic effects. Analyses, including colorimetric MTT assays and microscopic measurements of confluence, were conducted before and after treatment with dual system GP3 as a model (Figure 4). Cell viability assays were performed at various concentrations (3.125–100 µg/mL). Following treatment with single systems, such as samples GP3_PIP^−^ and GP3_TAZ, as well as dual system GP3_PIP^−^/TAZ, the affected cell lines were incubated for 72 h under standard conditions. Studies indicated that cytotoxicity increased with concentration, with cell viability dropping below 50% only at the highest concentration (100 μg/mL). Lower concentrations caused a slight decrease in viability, and the lowest tested concentration (3.125 μg/mL) did not significantly induce cell death. Notably, dual systems exhibited lower cytotoxicity compared with their single counterparts. Higher concentrations of polymer systems containing the drugs demonstrated increased cytotoxicity, while lower concentrations showed no significant differences compared with the action of free drugs against the BEAS-2B cell line. The confluence of cells treated with the tested compounds after 72 h of incubation was evaluated by comparing them to untreated control cells. The addition of systems with PIP^−^ did not cause any significant changes, or it even led to an increase in confluence, while TAZ incorporation induced a decrease in cell coverage (Figure 5). Microscopic images are presented in Figure 6.

## 4. Conclusions

Graft copolymers based on [2-(methacryloyloxy)ethyl]trimethylammonium chloride (TMAMA) units were employed as single-drug carriers, namely, conjugates with ionic piperacillin (PIP) and micelles loaded with tazobactam (TAZ), and dual-drug carriers with ionically conjugated piperacillin and encapsulated tazobactam for co-delivery applications. The polymers exhibited the capability to exchange chloride ions with PIP, resulting in a DC in the range of 45–73%. The amphiphilic properties of TMAMA-based graft copolymers, as estimated according to CMCs (values ranging from 0.011 to 0.073 mg/mL), proved advantageous for TAZ encapsulation in both single and dual systems (DLCs of 37–70% and 50–80%, respectively). The self-assembling copolymers with encapsulated TAZ formed particles of smaller sizes in dual-drug systems (24–192 nm) compared with single ones (97–319 nm). In vitro release studies on the single-drug systems revealed 66–81% of free PIP (7.8–15.0 μg/mL) and 47–98% of free TAZ (7.5–9.0 μg/mL) after 48 h. In dual-drug co-delivery systems bearing both PIP^−^ and TAZ, there was an inhibitory effect on the release of ionically bound PIP (21–25%, 2.6–3.9 μg/mL), while it did not influence the release of non-ionic TAZ (achieving 47–69%). However, due to high DLC values, the concentrations of free TAZ were improved (9.6–10.4 μg/mL). In vitro cytotoxicity studies indicated a negligible effect on cell viability at low concentrations. Therefore, these designed polymers show promise as carriers for the conjugation and/or encapsulation of PIP and TAZ, serving as co-delivery systems for the simultaneous release of two drugs.

## Figures and Tables

**Table 1:** Data for TMAMA-based graft copolymers [31].

No.	n_sc_	DG (mol. %)	DP_sc_	F_TMAMA_ (mol. %)	M_n_ × 10^−3^ (g/mol)	M_w_/M_n_
GP1	48	26	35	39	273.1	1.15
GP2	133	46	28	36	583.5	1.03
GP3			65	18	1090.5	1.11

n_sc_—the number of side chains; DG—the degree of grafting, equal to n_sc_ per total DP_n_, where DP_n,GP1_ = 186 and DP_n,GP2,GP3_ = 292; F_TMAMA_—the content of TMAMA in the side chains; M_n_—average number of molecular weight; M_w_/M_n_—molecular weight distribution.

**Table 2:** Characteristics of choline-based copolymers with Cl and after exchange with PIP anions using the goniometric method.

No.	CMC ^a^ (mg/mL)		Contact Angle ^b^ (°)	
	Cl (23)	PIP	Cl (23)	PIP
GP1	0.013	0.073	56.3	36.1
GP2	0.020	0.041	48.9	35.5
GP3	0.011	0.044	44.3	44.3

CMC: critical micelle concentration; ^a^ measured through IFT at a series of concentrations; ^b^ measured using sessile drop method on the polymer film (0.3 mg/mL polymer solution spin-coated on glass plate).

**Table 3:** Hydrodynamic diameters of PIL-based particles determined using DLS.

	TAZ			PIP^−^			PIP^−^/TAZ		
	D_h_ (nm)	Fraction (%)	PDI	Dh (nm)	Fraction (%)	PDI	Dh (nm)	Fraction (%)	PDI
GP1			0.004	20	77.8	0.26	24	42.4	0.36
	319	100		244	11.7		192	55.2	
				1714	10.5		1875	2.4	
GP2	16	24.4	0.17				26	67.1	0.42
	97	41.3							
	504	24.9					83	32.9	
	5430	9.4							
GP3	247	97.4	0.09	31	68.3	1.15	48	54.3	1.06
	6000	2.6		451	31.7		770	45.7	

**Table 4:** Drug release data for single and dual systems GP1–GP3.

	Single System		Dual System		Single System		Dual System	
	TAZ		TAZ		PIP		PIP	
	%	C (μg/mL)	%	C (μg/mL)	%	C (μg/mL)	%	C (μg/mL)
GP1	47	8.30	47	9.60	79	10.81	25	3.41
GP2	68	7.52	69	10.41	66	7.76	23	2.64
GP3	98	9.04	64	8.27	81	15.04	21	3.94

**Table 5:** Correlation coefficients (R^2^) and drug release exponents (n) of kinetic model equations.

Drug	Type	Matrix	First-Order Model	Higuchi Model	Korsmeyer–Peppas Model	
			R^2^	R^2^	R^2^	n
TAZ	Single	GP1	0.88	0.89	0.87	0.35
		GP2	0.96	0.98	0.99	0.60
		GP3	0.77	0.96	0.96	0.48
	Dual	GP1	0.83	0.84	0.77	0.46
		GP2	0.88	0.91	0.92	0.38
		GP3	0.93	0.94	0.79	0.80
PIP^−^	Single	GP1	0.99	0.98	0.94	0.29
		GP2	0.94	0.98	0.98	0.36
		GP3	0.95	0.97	0.99	0.38
	Dual	GP1	0.91	0.94	0.90	0.48
		GP2	0.90	0.97	0.97	0.64
		GP3	0.91	0.99	0.95	0.56

## Data Availability

The original contributions presented in the study are included in the article/supplementary material; further inquiries can be directed to the corresponding author/s.

## Supplementary Materials

The following supporting information can be downloaded at: https://www.mdpi.com/article/10.3390/pharmaceutics16020198/s1, Figure S1: DLS histograms for nanoparticles based on (a) GP1, (b) GP2, and (c) GP3 copolymers, Figure S2: Kinetic profiles according to models of first order, Higuchi, and Korsmeyer–Peppas for release of TAZ from (a) single systems and (b) dual systems, as well as PIP release from (c) single systems and (d) dual systems based on the grafted copolymers.
