# Supplementary material for: Piperacillin/Tazobactam Co-Delivery by Micellar Ionic Conjugate Systems Carrying Pharmaceutical Anions and Encapsulated Drug
Source: Pharmaceutics. 2024 Jan 30;16(2):198. doi: 10.3390/pharmaceutics16020198 (PMC10891911; doi:10.3390/pharmaceutics16020198)
Supplement: Supplementary file 1 [file pharmaceutics-16-00198-s001.zip › pharmaceutics-2807338-supplementary.pdf]

Supplementary Materials

Piperacillin/Tazobactam Co-Delivery by Micellar Ionic Conjugate Systems Carrying Pharmaceutical Anions and Encapsulated Drug

Katarzyna Niesyto, Aleksy Mazur and Dorota Neugebauer *

Department of Physical Chemistry and Technology of Polymers, Faculty of Chemistry, Silesian University of Technology, 44-100 Gliwice, Poland; katarzyna.niesyto@polsl.pl (K.N.); aleksy.mazur@polsl.pl (A.M.)

* Correspondence: dorota.neugebauer@polsl.pl

Content:

Figure S1. DLS histograms for nanoparticles based on a) GP1, b) GP2, and c) GP3 copolymer.

Figure S2. Kinetics profiles by models of first order, Higuchi and Korsmeyer-Peppas for release of TAZ from a) single and b) dual systems; as well as PIP release from c) single and d) dual systems based on the grafted copolymers.

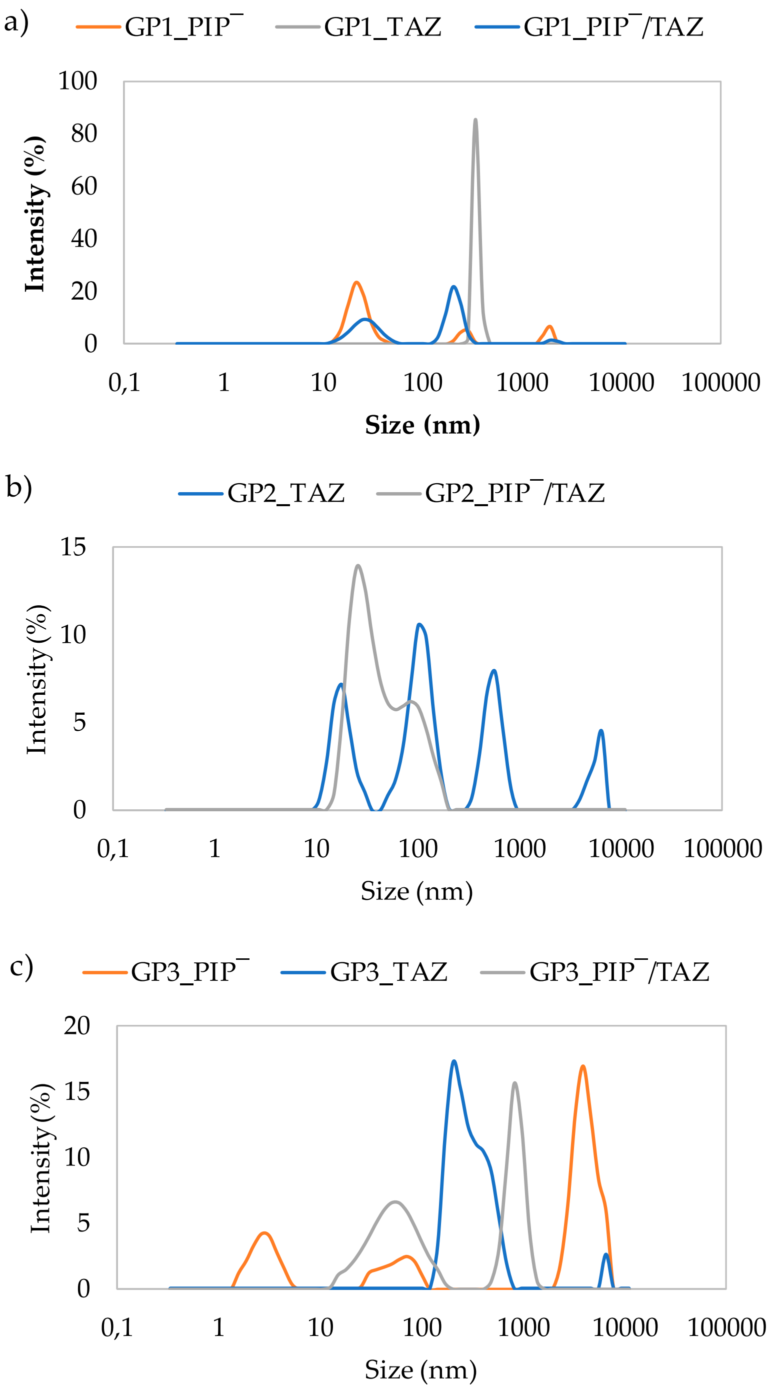

Figure S1. DLS histograms for nanoparticles based on a) GP1, b) GP2, and c) GP3 copolymer.

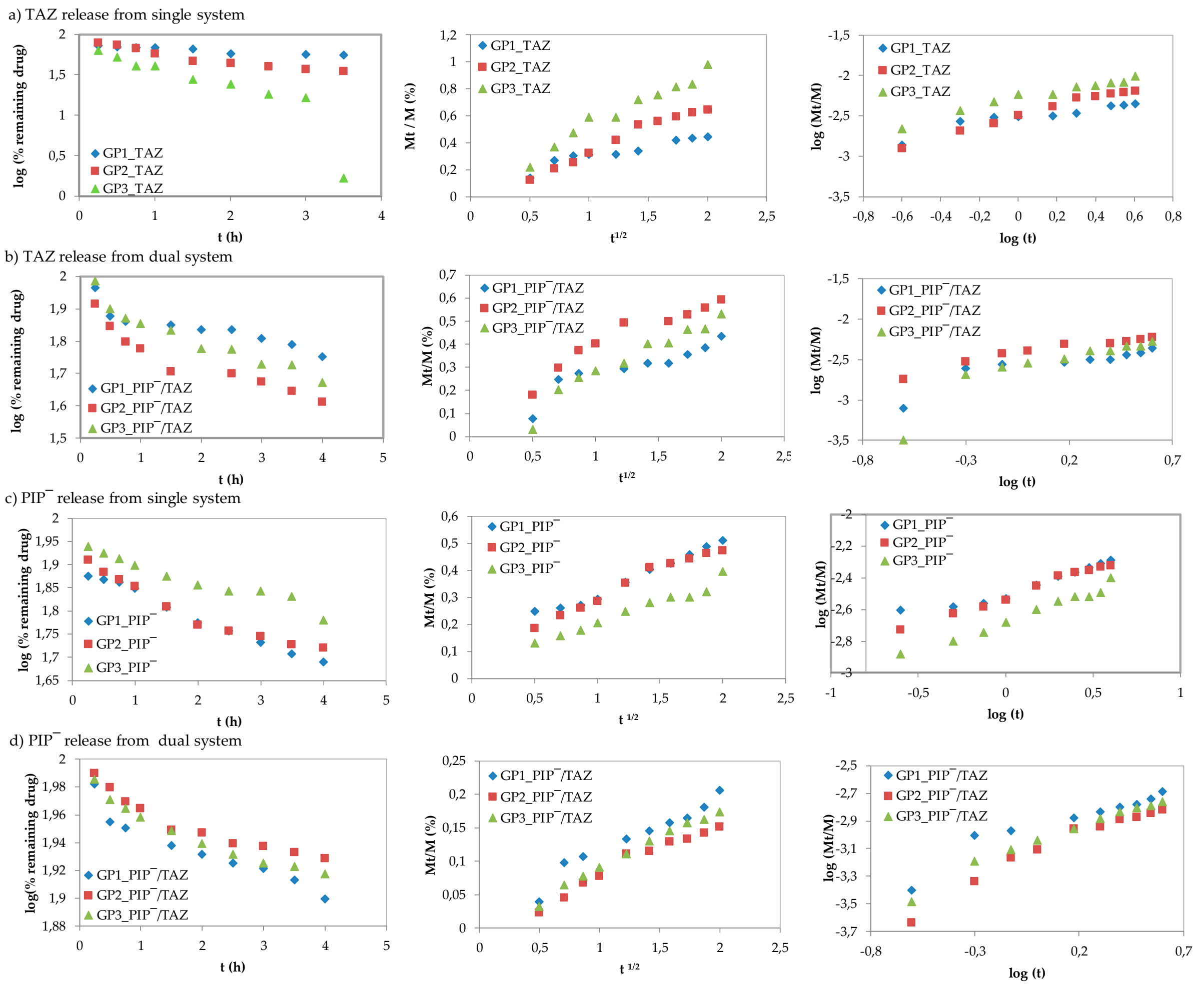

Figure S2. Kinetics profiles by models of first order (left column), Higuchi (central column) and Korsmeyer-Peppas (right column) for release of TAZ from a) single and b) dual systems; as well as PIP release from c) single and d) dual systems based on the grafted copolymers.
